# Structural brain abnormalities in cervical dystonia

**DOI:** 10.1186/1471-2202-14-123

**Published:** 2013-10-16

**Authors:** Tino Prell, Thomas Peschel, Bernadette Köhler, Martin H Bokemeyer, Reinhard Dengler, Albrecht Günther, Julian Grosskreutz

**Affiliations:** 1 Hans-Berger Department of Neurology, Jena University Hospital, Erlanger Allee 101, 07747 Jena, Germany; 2 Department of Psychiatry and Psychotherapy, Medical School Hannover, Carl-Neuberg-Strasse 1, 80625 Hannover, Germany; 3 Department of Neurology and Clinical Neurophysiology, Medical School Hannover, Carl-Neuberg-Str.1, 30625 Hannover, Germany; 4 Department of Neuroradiology, Jena University Hospital, Erlanger Allee 101, 07747 Jena, Germany; 5 Center for Sepsis Control and Care, Jena University Hospital, Erlanger Allee 101, 07747 Jena, Germany

**Keywords:** Visual system, Basal ganglia, Dorsolateral prefrontal cortex

## Abstract

**Background:**

Idiopathic cervical dystonia is characterized by involuntary spasms, tremors or jerks. It is not restricted to a disturbance in the basal ganglia system because non-conventional voxel-based MRI morphometry (VBM) and diffusion tensor imaging (DTI) have detected numerous regional changes in the brains of patients.

In this study scans of 24 patients with cervical dystonia and 24 age-and sex-matched controls were analysed using VBM, DTI and magnetization transfer imaging (MTI) using a voxel-based approach and a region-of-interest analysis. Results were correlated with UDRS, TWSTRS and disease duration.

**Results:**

We found structural alterations in the basal ganglia; thalamus; motor cortex; premotor cortex; frontal, temporal and parietal cortices; visual system; cerebellum and brainstem of the patients with dystonia.

**Conclusions:**

Cervical dystonia is a multisystem disease involving several networks such as the motor, sensory and visual systems.

## Background

Idiopathic cervical dystonia, the most common form of focal dystonia [1], is characterized by involuntary posturing of the head caused by involuntary spasms, tremors or jerks, and it is frequently accompanied by neck pain. Typical patterns are the rotational torticollis, laterocollis and retro- or antecollis [2]. Diagnosis is based on history, neurological examination and exclusion of other causes. Functional and structural neuroimaging techniques have detected regional activation and structural changes in the brains of patients with cervical dystonia.

Till date, 5 studies have used voxel-based morphometry (VBM) as a fully automated, operator-independent, whole-brain image analysis technique to evaluate cervical dystonia. They revealed an increase in grey matter volume in the internal globus pallidus, thalamus, cerebellum, motor cortex and supplementary motor area. However, the results are conflicting because a decrease in grey matter was also observed in the putamen, supplementary motor area, right visual cortex and right dorsal lateral prefrontal cortex [3-6].

Diffusion tensor imaging (DTI), which is more sensitive to pathology in white matter fibres, detects alterations in the degree (diffusivity, ADC) and directedness (fractional anisotropy, FA) of proton movement, which reflect microstructural tissue changes and fibre organisation. Several studies have examined DTI measures in patients with different forms of dystonia. Similar to VBM studies, the results of DTI studies are contradictory. In patients with hereditary dystonia, FA was decreased in the sensorimotor cortex [7]. In an ROI-based study of patients with dystonia, FA was decreased in the corpus callosum and increased in the putamen [8,9]. Diffusivity was enhanced in the prefrontal cortex and decreased in the caudate nucleus and putamen [9]. In contrast, in another ROI study, diffusivity in the pallidum, putamen and caudate nucleus was increased in patients with cervical dystonia [6,10].

Magnetization transfer imaging (MTI) detects the relative proportion of free mobile protons and immobile protons bound to macromolecules, i.e. in myelin and axonal loss [11,12], and it has not yet been applied to patients with dystonia.

We aimed to use a voxel-based analysis and an ROI-based approach to evaluate idiopathic cervical dystonia and extend previous MRI studies. Voxel-based analysis is a technique that can identify microstructural changes in any part of the whole brain without a prior hypothesis, unlike ROI-based studies. We therefore studied a moderate sample of 24 patients with dystonia using VBM, DTI and MTI.

## Methods

### Subjects

Twenty-four patients (18 females, 6 males) with idiopathic cervical dystonia (mean age, 52 years) and 24 age- and sex-matched controls (18 females, 6 males; mean age, 53 years, p > 0.05) from the Medical School Hannover were enrolled in this prospective study from July 2004 until March 2005. Patients who were diagnosed with idiopathic cervical dystonia by a neurologist experienced in the diagnosis of dystonia were included. Exclusion criteria were as follows: presence of any multifocal dystonia with blepharospasm or oromandibular manifestations, a positive family history for dystonia and a history of any other neurological disease or relevant vascular diseases. Every patient was regularly treated with botulinum toxin, and the last injection was administered 4 weeks before MRI imaging. Patients were evaluated using the Unified Dystonia Rating Scale (UDRS) and Toronto Western Spasmodic Torticollis Rating Score (TWSTRS). Using the Mini-Mental Status Examination (MMSE) and the Beck Depression Inventory (BDI), clinically relevant dementia or depression was ruled out (Table 1). Exclusion criteria for healthy voluntary controls were: history of neurological or psychiatric disorders, vascular diseases (e.g. stroke), diabetes mellitus or the intake of psychoactive drugs. This study was approved by the local ethics committee of the Hannover Medical School, and all subjects provided written informed consent.

**Table 1:** Disease characteristics of probands

	**Sex**	**Age**	**UDRS**	**TWSTRS**	**Disease duration**	**BDI**	**MMST**
	**(f/m)**	**(y)**			**(y)**		
**Patients**	18/6	52	4.3	22.5	13.9	7,5	28.3
**Controls**	18/6	53	-	-	-	-	29.1

### Data acquisition

Images were acquired on a neuro-optimized 1.5-T GE Signa Horizon NV/i (General Electric Company, Milwaukee, WI, USA) using a 3-dimensional, T1-weighted, spoiled gradient-recalled echo (SPGR) sequence generating 124 contiguous sagittal slices (RT, 24 ms; TE, 8 ms; flip angle, 180°; 2 averages; acquisition time, 13′10”; in-plane resolution, 0.97 × 0.97 × 1.5 mm^3^). The protocol for MTI included a proton density (PD)-weighted spin echo sequence (TR, 2600; TE, 20; 256 × 256), both with (MT) and without (non-MT) a preparing saturation pulse (1200 Hz off-resonance, 1180° flip-angle, 16 ms). Forty-three slices of 3-mm thickness aligned along the AC–PC line were acquired. Image post-processing included a simple intersequence correction of movement with the automated image registration package based on the rigid body model (AIR) [13] and calculation of MTR maps pixel-by-pixel according to the following formula: MTR = [(non-MT − MT)/non-MT] × 100. During scanning, all participants were comfortably placed, and their heads were fixated within the headcoil with special cushions.

### Pre-processing of structural data

Data were processed on a standard IBM-compatible PC using SPM2 statistical parametric mapping software (Welcome Department of Cognitive Neurology, London), and they were worked on in an analysis environment (MATLAB; the Math Works Inc, Natick, Mass). The images were re-oriented into oblique axial slices aligned parallel to the anterior–posterior commissural axis with the origin set to the anterior commissure.

### Data pre-processing for VBM

An optimized version of the VBM protocol was followed as described by our group [14]. The resulting images were re-sliced to a final voxel size of 1 mm^3^. Voxel values in segmented images of grey and white matter were multiplied by the Jacobian determinants derived from spatial normalization to provide an intensity correction for induced regional volumetric changes, thus preserving within-voxel volumes that may have been altered during non-linear normalization. These ‘modulated’ images were smoothed to 8 mm using a full width half-maximum (FWHM) Gaussian filter to minimize individual gyral variations and increase the statistical validity of the analysis.

### Data pre-processing for voxel-based DTI

After calculation of the FA and ADC maps, images were pre-processed using an approach adopted from VBM as described by our group [14]. This included an optimized normalization procedure, automated removal of skull and CSF signals as well as smoothing (8-mm FWHM). All EPI scans were normalized to the EPI template provided by SPM and further used to create a site-specific EPI template appropriate to the population sample with scanner-specific image contrast. This site-specific template was used again for normalization and brain extraction for the individual images in the group studied, thus resulting in optimal normalization and cleaning parameters for use with the FA and ADC images.

### Data pre-processing for voxel-based MTI

The main challenge of voxel-based MTI analysis involves meeting the requirement for an optimal matching of the brains being compared. Therefore, a complex pre-processing procedure was employed as previously described [15]; this procedure included the creation of a series of templates in order to derive the best possible parameter set for normalization. To achieve maximum precision, a proton density (PD)-weighted template with scanner-specific image contrast best adapted for the studied sample population was created. Therefore, all PD-weighted scans were normalized to the PD template provided by SPM2 and subsequently smoothed with an 8-mm isotropic FWHM isotropic Gaussian kernel. Therefore, a mean image was created that served in the following as the sample population and scanner specific template, and all PD-images in native space were normalized to it. To minimize the influence of structures other than the brain on registration, the normalized images were skull-stripped using the segmentation procedures implemented in SPM2, which included the brain extraction step. The resultant white and grey matter partitions were summed and set at a threshold of 0.15, thus discarding the majority of extracerebral tissue and CSF while preserving the original voxel intensities. The obtained normalized skull-stripped images were then smoothed with an 8-mm isotropic FWHM Gaussian kernel, and a new skull-stripped PD-weighted template was created. The PD images in native space (non-normalized) were subject to exactly the same procedure, wherein they were skull-stripped, segmented, multiplied with a binary mask and normalized to the previously skull-stripped PD-weighted template. This prevented any contribution of non-brain voxels, afforded optimal spatial normalization of the individual PD images and provided an optimized normalization parameter set for the PD images adapted to the investigated population sample. Because the segmentation of native images is performed on affine-normalized images, and because the probability maps used as Bayesian priors for segmentation are in stereotactic space, the optimized normalization parameter set was re-applied to the original PD-weighted images in native space. These optimally normalized PD images—now in stereotactic space—were again skull-stripped using the above described procedure. This resulted in optimally normalized PD images removed from extracerebral tissue and CSF. Finally, the optimized normalization parameter set was applied to the inherently co-registered MTR maps in native space and re-sliced with a final voxel size of 1 mm^3^. The normalized MTR images were then skull-stripped by applying the corresponding brain mask derived from the optimally normalized PD-weighted images. Analogous to the PD images, this resulted in optimally normalized MTR maps removed from non-brain structures. Images were smoothed to 8 mm using an FWHM Gaussian filter to improve signal-to-noise ratio.

### Statistical analysis

Processed images of each tissue class were analyzed in the framework of the general linear model. For VBM, group comparison of patients with dystonia and healthy controls was performed in SPM2 using the model ‘compare-populations, one scan/subject (ANCOVA)’. During modulation, we incorporated the correction for volume change induced by spatial normalization. Therefore, it was appropriate to include the global mean voxel value of each tissue class as a covariate to determine the regionally specific pattern of loss or gain within each compartment and eliminate any variance due to differences in head size. Regression analyses with clinical measures were explored for the patients using the SPM2 model ‘simple regression (correlation)’. With regard to group comparisons, ANCOVA with the mean voxel value was used to normalize image intensity in the different tissue maps to allow for identification of the regional pattern of these correlations. MTR maps were analyzed separately using the model ‘compare-populations, one scan/subject (two sample *t*-test)’. Regression analyses with clinical measures in the patients were explored using the model ‘simple regression (correlation)’. The resulting statistical parametric maps of VBM and MTR were derived at a significance level of p < 0.001, uncorrected with a threshold of 100 voxels.

In the group comparison, a small volume correction (SVC) limited to the volume of that particular region was performed for regions in which an effect was hypothesized, namely the globus pallidum and visual cortex [3-5,16], using a sphere with a radius of 15 mm [17]. Here we controlled for multiple comparisons using the family-wise error (FWE) method (p < 0.05).

Moreover, for the group comparison, region of interest (ROI) analyses were performed using MarsBar (MARSeille Boîte À Région d’Intérêt; release 0.43) and the AAL ROI archive, which is a toolbox for SPM providing routines and an ROI library (http://marsbar.sourceforge.net/). The ROIs were as follows: cerebellum, occipital lobe, para/hippocampal region, frontal lobe, parietal lobe, temporal lobe and precuneus. The ROI definitions are described in the study by Tzourio-Mazoyer et al. [18].

## Results

### Clinical characterization

Twenty-four patients with dystonia (mean age 52 years) were enrolled in this study. The mean disease duration was 13.4 years (SD 10.4) and the mean age at disease onset was 37.1 years (SD 15.2). The mean TWSTRS was 22.5 points (SD 10.7). The subscores of TWSTRS were as follows. First, the mean Torticollis Severity Scale was 9.5 (SD 5.2). Maximal Excursion was rotation and laterocollis, and none of the patients had antercollis. Most of the patients had an intermittent (25%–50% of the time) or a frequent deviation (50%–75%). Sensory tricks were completely or partially effective in 87.5% patients. Sixty-seven percent patients were able to maintain their head within 10° of the neutral position without using sensory tricks for over 46 seconds. Second, the Mean Disability Scale was 6.2 points (SD 5.2). Third, the Mean Pain Scale was 6.4 (SD 3.7). The mean severity of pain was 3.2, calculated as [worst + best + (2*usual)/4]. On an average, pain was present 26%–50% of the time, and it was a major contributor to disability in 42% patients. The mean UDRS was 4.25 (SD 4.1). The clinical parameters are summarized in Table 1.

### Group comparison

#### VBM

**Figure 1:**
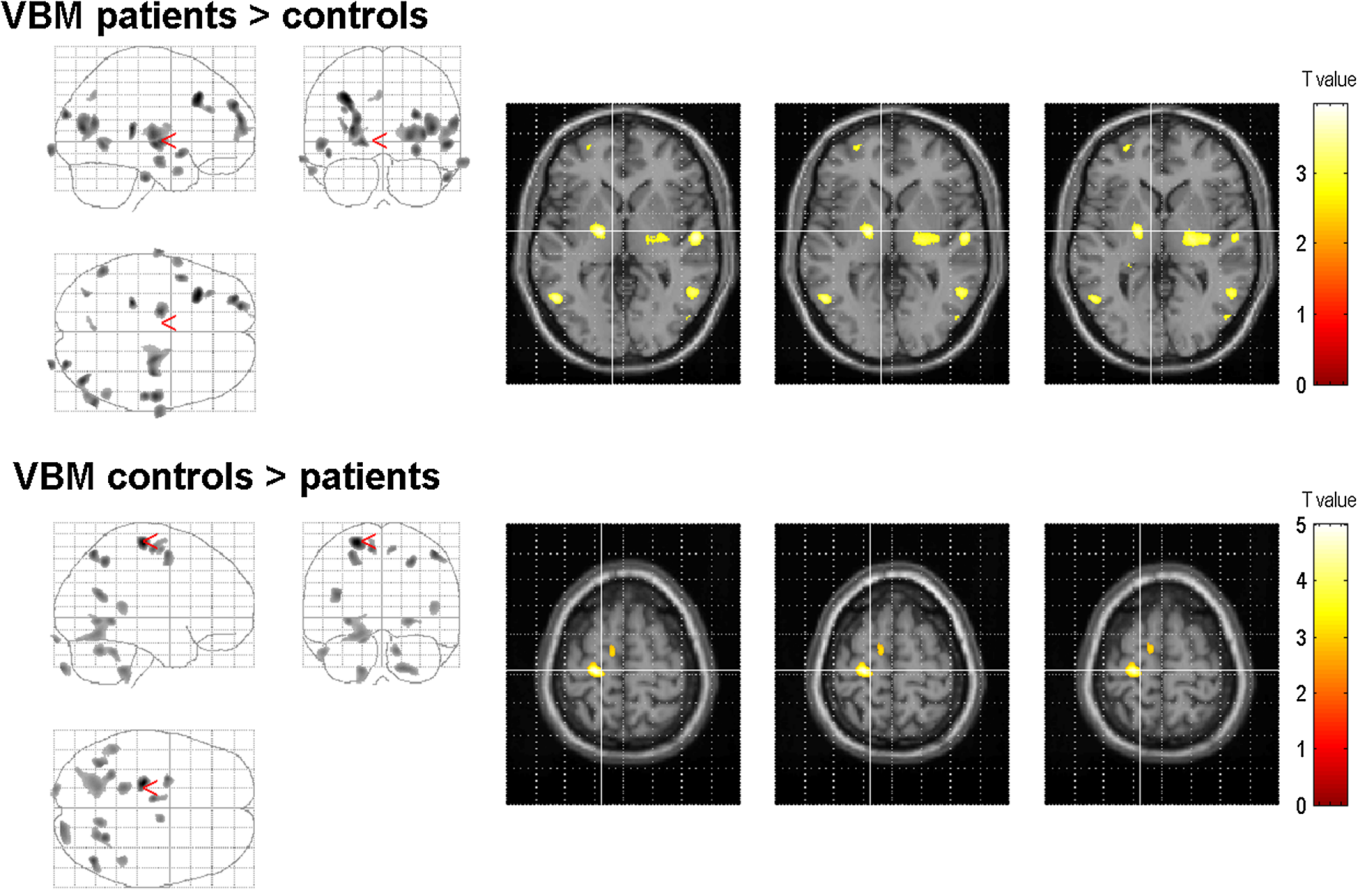
**Changes of grey matter volume in patients with dystonia compared to those in healthy controls (group comparison).** VBM showed regional enhanced grey matter in the lentiform nucleus, the frontal eye fields (BA 8), and the secondary visual cortex (displayed at p = 0.001, uncorrected, extent threshold 100 voxels). Grey matter atrophy was observed in the precentral gyrus, the supplementary motor area, the somatosensory association cortex, and in the medial temporal gyrus. The color bar represents the T-score. The differences between the groups are superimposed on a standard normalized T1-weighted image. Images are shown in neurological convention.

In the voxel-based whole brain analysis, the dystonia patients displayed enhanced volume bilaterally in the lentiform nucleus (left globus pallidus, right claustrum, right putamen), left frontal eye field (BA 8) and the bilateral medial surface of the occipital lobe (visual cortex, BA 17) (Figure 1). In addition, there were regions with decreased grey matter volume, particularly in the left precentral gyrus, the left supplementary motor area (BA 6), the right somatosensory association cortex (BA 7) and the left medial temporal gyrus (Table 2, Figure 1). In the white matter tissue class and CSF, no significant voxels were found.

**Table 2:** Changes of grey matter volume, MTI, and DTI-values in patients with dystonia compared to those in healthy controls (group comparison)

**Brain region**	**Side**	**BA**	**MNI space**			**t-value**	**P (uncorr.)**	**Cluster size**
			**x**	**y**	**z**			
**VBM**								
**Grey matter volume increase**								
Frontal gyrus	left	8	−32	23	38	4.64	0.002	582
Frontal gyrus	left	10	−29	55	32	4.01	0.009	464
			−22	60	19	4.02		
			−23	61	14	3.68		
Medial globus pallidus (internal)	left	-	−17	−9	0	3.99	0.011	147
Claustrum, Putamen	right	-	35	−16	7	3.90	0.014	640
			24	−15	7	3.83		
Middle temporal gyrus	right	39	42	−76	12	4.06	0.009	454
Occipital lobe, cuneus	right	19	28	−90	23	4.07	0.031	118
Occipital lobe, cuneus	left	19	−53	−58	−3	3.71	0.022	149
**Grey matter volume decrease**								
Precentral gyrus	left	4	−22	−23	65	5.03	0.001	512
Middle frontal gyrus	left	6	−25	−2	54	3.83	0.016	188
			−8	−14	59	3.87	0.015	100
Parietal Lobe. Precuneus	right	7	26	−56	49	3.93	0.013	167
Middle temporal gyrus	left	39	−38	−60	20	3.78	0.018	192
**MTI**								
**Increase of MTR**								
Middle temporal gyrus	left	39	−47	−69	28	5.02	0.025	1854
		19	−51	−61	17	3.95		
Temporal lobe	right	21	42	−4	−23	4.47	0.004	2676
			49	−19	−17	4.36		
Inferior parietal lobule	left	13	−58	−42	22	4.24	0.006	485
Cingulate gyrus	left	31	−6	−47	42	3.86	0.016	104
**Decrease of MTR**								
Frontal gyrus	left	11	−1	58	−17	4.4	0.004	450
		10	−35	38	26	4.23	0.006	612
Precentral gyrus	left	9	−36	23	39	4.10	0.009	423
Lingual gyrus	left	17	−15	−95	−12	4.17	0.007	730
Middle temporal gyrus	right	19	40	−75	18	4.14	0.008	246
**DTI**								
**Increase of FA**								
Middle frontal gyrus	left	6	−3	−16	62	4.05	0.012	149
Brainstem. Pons	left	-	−13	−29	−29	3.94	0.016	213
Thalamus (Ncl. VPN, VPL)	left	-	−9	−24	−3	3.91	0.017	319
Middle temporal gyrus	left	39	−40	−62	18	4.92	0.001	481
Posterior cingulate gyrus	right	-	18	−58	14	3.66	0.031	103
**Decrease of FA**								
Precentral gyrus	right	6	50	1	33	4.85	0.001	481
Postcentral gyrus	left	3	−38	−25	50	4.23	0.008	582
Middle frontal gyrus	left	9	−32	22	36	5.14	0.001	950
			−37	32	28	4.00	0.014	163
Inferior parietal lobule	right	40	42	−62	46	5.25	<0.001	830
Parietal lobe, cuneus	right	-	25	−74	32	3.85	0.020	129
Middle temporal gyrus	right	37	58	−54	−2	4.00	0.014	144
Middle temporal gyrus	right	19	40	−76	18	3.89	0.018	269
**Increase of ADC**								
Middle temporal gyrus	left	39	−55	−66	16	4.76	0.017*	1777
			−45	−77	32	3.48		
Occipital lobe, cuneus	right	18	18	−102	6	5.01	0.001	726
Superior parietal lobule	right	7	34	−63	59	4.31	0.006	435
			16	−72	57	4.06	0.011	239
Superior parietal lobule	left	7	−30	−49	42	4.18	0.008	117

* significant with FDR (false discovery rate).

ROI analysis displayed an increase in grey matter volume in the visual system on the lateral surface of the occipital lobe (superior and middle occipital gyri), the lateral and medial surface of the parietal lobe (supramarginal and angular gyri, precuneus) and the limbic system (hippocampal and parahippocampal regions; Table 3). Decreased grey matter volume was not observed in the studied ROIs.

**Table 3:** ROI analyses of grey matter volume, MTI, and DTI-values in patients with dystonia compared to those in healthy controls (group comparison)

**ROI**		**t-value**	**P (uncorr.)**
**VBM**			
**Grey matter volume increase**			
Angular gyrus	right	2.75	0.004
Cerebellum	right	1.70	0.047
Inferior frontal gyrus, orbital part	right	1.81	0.038
Hippocampus	left	2.06	0.022
Middle occipital gyrus	right	2.32	0.012
Superior occipital gyrus	left	1.74	0.044
Superior occipital gyrus	right	2.43	0.009
Parahippocampal gyrus	left	2.23	0.015
Parahippocampal gyrus	right	1.93	0.029
Inferior parietal gyrus	left	2.13	0.019
Inferior parietal gyrus	right	2.25	0.014
Superior parietal gyrus	left	2.90	0.002
Superior parietal gyrus	right	2.81	0.003
Postcentral gyrus	left	2.15	0.018
Postcentral gyrus	right	1.58	0.060
Precentral gyrus	right	1.76	0.042
Precuneus	left	2.39	0.010
Precuneus	right	1.73	0.044
Supramarginal gyrus	right	2.07	0.021
Middle temporal gyrus	right	2.23	0.015
Cerebellum, vermis		1.77	0.041
**Grey matter volume decrease**			
none			
**MTR**			
**Increase of MTR**			
Angular gyrus	right	2.75	0.004
Cerebellum 3	right	1.70	0.047
Inferior frontal gyrus, orbital part	right	1.81	0.038
Hippocampus	left	2.06	0.022
Middle occipital gyrus	right	2.32	0.012
Superior occipital gyrus	left	1.74	0.044
Superior occipital gyrus	right	2. 43	0.009
Parahippocampal gyrus	left	2.23	0.015
Parahippocampal gyrus	right	1.93	0.029
Paracentral lobule	left	1.83	0.037
Paracentral lobule	right	2.06	0.022
Inferior parietal gyrus	left	2.13	0.019
Inferior parietal gyrus	right	2.25	0.014
Superior parietal gyrus	left	2.90	0.002
Superior parietal gyrus	right	2.81	0.003
Postcentral gyrus	left	2.15	0.018
Precentral gyrus	right	1.76	0.042
Precuneus	left	2.39	0.010
Precuneus	right	1.73	0.044
Supramarginal gyrus	right	2.07	0.021
Middle temporal gyrus	right	2.23	0.015
Cerebellum, vermis		1.77	0.041
**Decrease of MTR**			
none			
**ADC**			
**Increase of ADC**			
Angular gyrus	right	2.33	0.012
Superior occipital gyrus	right	2.05	0.023
Parahippocampal gyrus	left	2.12	0.019
Paracentral lobule	right	1.77	0.041
Inferior parietal gyrus	right	1.68	0.049
Superior parietal gyrus	left	2.65	0.005
Superior parietal gyrus	right	2.57	0.006
Postcentral gyrus	left	1.75	0.043
Precuneus	left	1.88	0.015
Middle temporal gyrus	right	2.22	0.049
**Decrease of ADC**			
Cerebellum, crus	left	2.13	0.019
Inferior frontal gyrus, opercular part	left	2.12	0.019
Inferior frontal gyrus, triangular part	left	1.91	0.030
Lingual gyrus	left	1.83	0.036
Putamen	right	2.00	0.025
**FA**			
**Increase of FA**			
Angular gyrus	right	2.75	0.004
Cerebellum	right	1.70	0.047
Inferior frontal gyrus, orbital part	right	1.81	0.038
Hippocampus	left	2.06	0.022
Superior occipital gyrus	left	1.74	0.044
Superior occipital gyrus	right	2.43	0.009
Parahippocampal gyrus	left	2.23	0.015
Parahippocampal gyrus	right	1.93	0.029
Paracentral lobule	left	1.83	0.037
Paracentral lobule	right	2.06	0.022
Inferior parietal gyrus	left	2.13	0.019
Inferior parietal gyrus	right	2.25	0.014
Superior parietal gyrus	left	2.90	0.002
Superior parietal gyrus	right	2.81	0.003
Postcentral gyrus	left	2.15	0.018
Precentral gyrus	right	1.76	0.042
Precuneus	left	2.39	0.010
Precuneus	right	1.73	0.044
Supramarginal gyrus	right	2.07	0.021
Middle temporal gyrus	right	2.23	0.015
Cerebellum, vermis		1.77	0.041
Cerebellum, vermis		1.96	0.028
**Decrease of FA**			
none			

#### MTI

**Figure 2:**
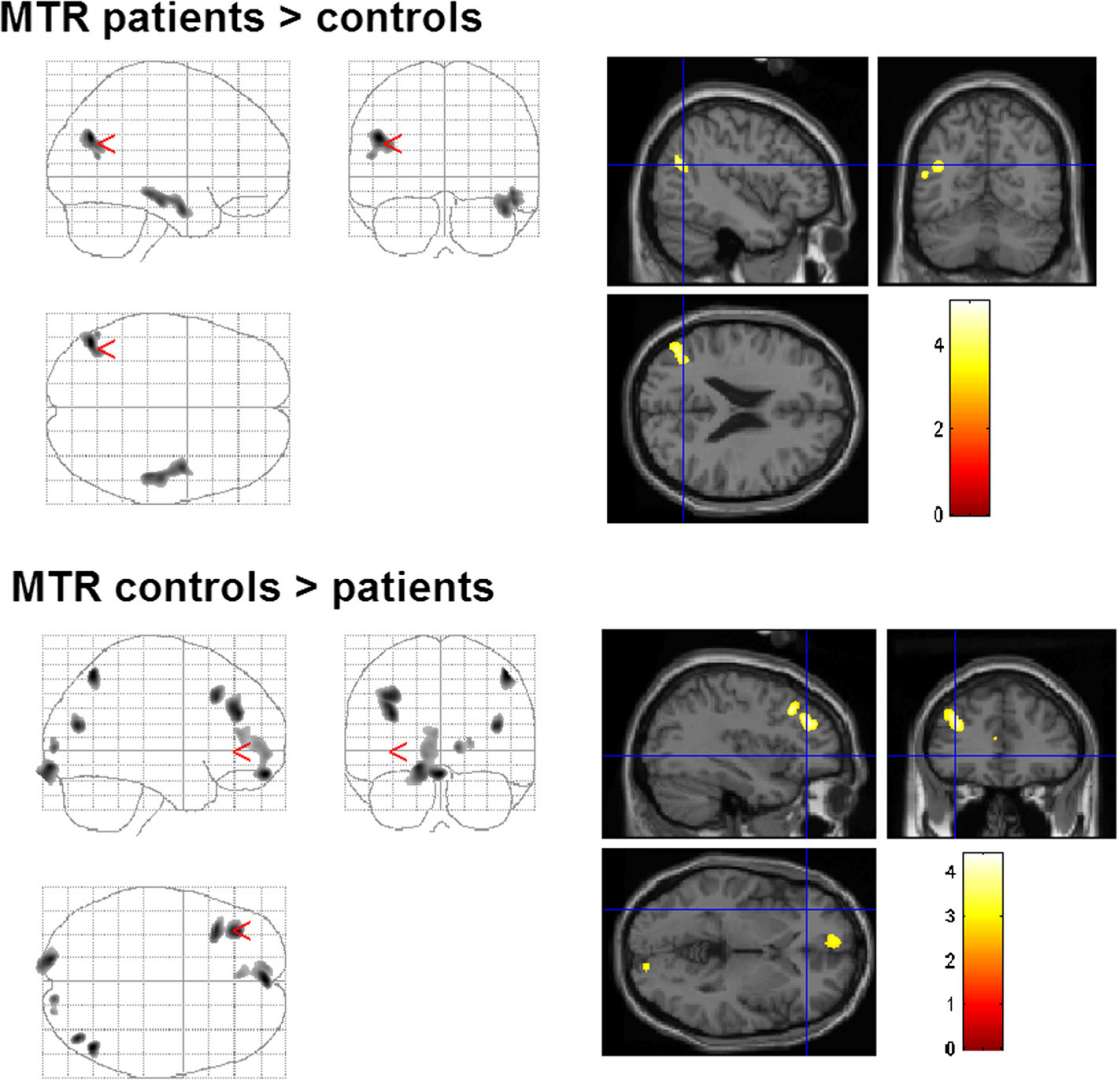
**Changes of MTR in patients with dystonia compared to those in healthy controls (group comparison).** Enhanced MTR was found in the left temporal medial gyrus, in the temporal lobe, in the secondary somatosensory cortex, and in the cingulate gyrus. MTR was reduced in the left dorsolateral prefrontal and frontal cortex (BA 9, 10, 11) and in the primary and secondary visual cortex (BA 17, 19).

In the voxel-based whole brain analysis, MTR was enhanced in the temporal (left medial temporal gyrus, right temporal lobe BA 21) and parietal (left secondary somatosensory cortex BA 13) lobe of the patients with dystonia. Furthermore, MTR was increased in the left cingulate gyrus. MTR was decreased in the left dorsolateral prefrontal and frontal cortex (BA 9, 10, 11) and the bilateral primary and secondary visual cortices (BA 17, 19; Table 2, Figure 2).

ROI analysis supported the involvement of the parietal lobe and, in particular, the inferior parietal lobule, by enhanced MTR. Similarly, in the lateral surface of the occipital lobe (superior and middle occipital gyri) and the limbic system (hippocampal and parahippocampal regions), MTR was found to be enhanced (Table 3). A decreased MTR was not observed in the studied ROIs.

#### DTI

**Figure 3:**
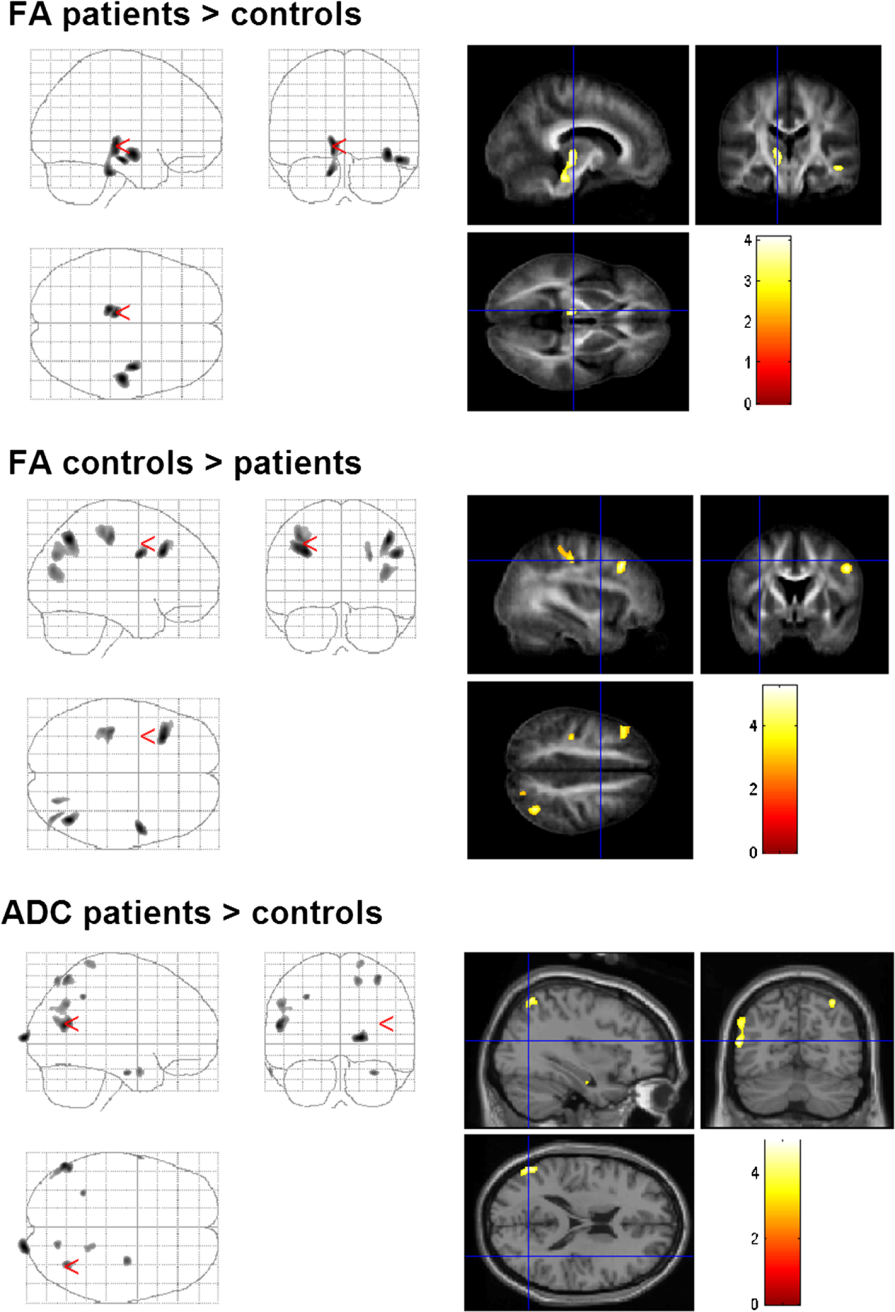
**Changes of DTI-values in patients with dystonia compared to those in healthy controls (group comparison).** FA was increased in brainstem, thalamus, and temporal areas. Decreased FA was observed in postcentral gyrus, frontal and occipital cortex. Diffusivity was enhanced in the area MT, and secondary somatosensory cortex.

FA values were changed in the motor, sensory, limbic and visual networks of the patients with dystonia. FA was enhanced in the supplementary motor area (middle frontal gyri, BA 6), pontine brainstem (medial lemniscus), thalamus (ventral posterior medial and lateral nucleus, VPM and VPL), middle temporal gyrus and cingulate gyrus (Table 2, Figure 3). FA was decreased in the precentral and postcentral gyri, dorsolateral prefrontal cortex and right secondary visual cortex (BA 19; Table 2, Figure 3).

Diffusivity was enhanced in the middle temporal gyrus (BA 19), medial surface of the occipital lobe (cuneus) and bilateral secondary somatosensory cortex (BA 7). Decreased diffusivity was not observed (Table 2, Figure 3).

ROI analysis showed enhanced FA in the parietal lobe and the limbic (hippocampal and parahippocampal regions) and visual systems (occipital lobe). A decreased FA was not observed. Similar to the whole brain approach, diffusivity was enhanced in the middle temporal gyrus (BA 19) and superior parietal lobule (Table 3).

### Correlation with clinical data

Next, patient’s clinical data such as disease severity and disease duration were correlated with VBM, MTI and DTI values. SVC and ROI analysis were not undertaken for studying these correlations.

The UDRS showed a clear positive correlation with grey matter decrease in the frontal areas, cingulate gyrus and inferior frontal gyrus, indicating that the progression of symptoms was reflected in an increase in grey matter volume in these areas. UDRS and MTR did not correlate significantly. With regard to DTI, UDRS showed a positive correlation with FA in the visual cortex (bilateral occipital lobe), cingulum and frontal and parietal lobes (BA 7). UDRS correlated negatively with ADC in the occipital lobe and cingulum (Table 4).

**Table 4:** Changes of grey matter volume, MTI, and DTI-values in patients with dystonia and their correlation with clinical data

**Brain region**	**Side**	**BA**	**MNI space**			**t-value**	**P (uncorr)**	**Cluster size**
			**x**	**y**	**z**			
**Positive correlation UDRS and grey matter volume**								
Frontal gyrus	right	11	1	22	−27	5.23	0.002	690
Cingulate gyrus	left	-	−12	−19	31	4.81	0.004	310
Inferior frontal gyrus	left	-	−45	27	6	3.97	0.022	134
**Positive correlation UDRS and FA**								
Middle occipital gyrus	right	19	36	−73	0	7.20	0.010*	1381
Cingulum, pars posterior	right	30	13	−65	8	5.00	0.001*	2258
Occipital lobe, cuneus								
		17	9	−83	5	4.73		
Occipital lobe	left	19	−40	−66	−16	5.02	0.006	200
		18	−2	−73	−6	4.70	0.011	495
Occipital lobe	left	19	−36	−83	4	4.27	0.024	416
			−27	−82	2	3.92		
Frontal lobe	left	10	−18	54	11	5.49	0.017*	1241
		32	−23	40	8	4.32		
		11	−23	42	−1	4.11		
Frontal lobe	left	8	−23	18	37	4.92	0.007	386
Frontal lobe	right	11	9	48	−16	4.72	0.010	161
Parietal lobe, precuneus	left	7	−13	−74	49	4.34	0.021	210
**Negative correlation UDRS and ADC**								
Occipital lobe, cingulum	right	-	19	−62	10	5.48	0.014*	1531
			20	−73	14	4.20		
			28	−71	18	4.02		
Middle occipital gyrus	right	19	38	−72	5	4.14	0.026	412
**Negative correlation disease duration and MTR**								
Middle frontal gyrus	left	6	−26	23	60	5.65	0.001	559
Precentral gyrus	left	4	−22	−28	64	4.99	0.002	549
Fusiform gyrus	right	37	46	−56	−21	4.97	0.002	687
**Negative correlation disease duration and FA**								
Superior frontal gyrus	left	8	−26	20	54	5.89	0.001	255
Parietal lobe, precuneus	left	7	19	−72	50	5.19	0.004	356
Parietal lobe, precuneus	right	7	−4	−72	44	4.32	0.022	135
Occipital lobe	left	17	−10	−92	−15	4.08	0.033	288
Temporal lobe	left	37	−44	−45	−13	4.15	0.029	278
			−40	−53	−10	3.79		
**Positive correlation disease duration and ADC**								
Occipital gyrus	right	18	30	−94	−5	4.53	0.012	430
			36	−91	−10	3.78		
Occipital lobe. cuneus	Right	-	5	−99	4	4.16	0.025	246
**Negative correlation disease duration and ADC**								
Middle frontal gyrus	left	8	−26	15	52	6.34	<0.001	335
		6	−41	−2	49	4.74	0.008	118
Middle frontal gyrus	right	6	25	−12	64	4.26	0.021	118
Inferior parietal lobule	right	40	40	−57	42	5.27	0.003	443
			46	−51	53	3.71		
Inferior parietal lobule	right	40	38	−40	43	4.43	0.015	154
Inferior parietal lobule	left	40	−50	−40	47	5.15	0.004	825
			−40	−41	43	4.66		
			−44	−33	42	4.03		
Postcentral gyrus	right	3	25	−31	54	5.34	0.003	172
Postcentral gyrus	left	3	−22	−30	63	4.80	0.008	614
Precentral gyrus	right	4	55	−10	45	4.55	0.012	382
		4	43	−14	59	4.24	0.022	283
Parietal lobe, precuneus	left	7	−16	−63	55	4.28	0.020	155

* significant with FDR (false discovery rate).

TWSTRS did not correlate with grey matter volume, MTR, FA and ADC.

The disease duration was not reflected in a specific pattern of volume decrease, but it correlated negatively with MTR in the primary motor cortex (BA 4), supplementary motor cortex (BA 6) and the right fusiform gyrus (BA 37). This indicates that neuronal density in the grey matter and myelinisation in the white matter decreases with increasing disease duration. The disease duration showed a negative correlation with FA in the temporal and parietal lobes, frontal eye field (BA 8) and the primary visual cortex. Disease duration and ADC correlated positively in the right visual cortex (BA 18) and negatively in the secondary somatosensory cortex (BA 40), precentral and postcentral gyri (BA 3, 4), supplementary motor cortex (BA 6) and the left frontal eye field (BA 8; Table 4).

## Discussion

In the present study, structural changes in the brains of a moderate sample of dystonia patients were investigated by VBM, DTI and MTI. We found widespread alterations in grey and white matter tissue in the basal ganglia and the somatosensory, motor, limbic and visual systems, providing further evidence that dystonia is a multisystem disease involving several brain networks. While some regions were found to be altered with only one method, consistent microstructural changes were observed with both the voxel-based whole brain approach and ROI analysis in the occipital, frontal and limbic lobes and the basal ganglia.

### Basal ganglia system

In the basal ganglia system, the striatum and the subthalamic nucleus are the two principal input nuclei that receive inputs from the cerebral cortex, limbic system and the thalamus, whereas the internal segment of the globus pallidus and substantia nigra pars reticulata provide outputs to the thalamus and brainstem [19]. Dystonia is regarded as a circuit disorder with aberrant activity within the basal ganglia and efferent connections of the basal ganglia with the thalamus and brainstem [20]. The hyperkinetic movement is probably caused by a loss of inhibition in motor control or, in the classic view, a relative overactivation of the direct pathway (striatum–internal globus pallidus–thalamus) [21]. Our study supports this view because we found structural changes in the basal ganglia, thalamus and brainstem.

We found a significant increase in grey matter in the putamen and pallidum. However, ROI analysis of both structures revealed no grey matter differences between the patients with dystonia and controls. Miscellaneous studies demonstrated grey matter increases in the putamen and internal globus pallidus [5,22,23]. However, these results are in contrast to a decrease in grey matter volume in the putamen in a smaller cohort of patients with dystonia [4] and in an ROI-based VBM study [16]. The discrepancy within all published morphometric studies on dystonia may be due to several methodological factors, among which the number of patients, disease duration and methods used for data analysis are the most important.

In another ROI-based analysis of the basal ganglia system, an increased FA in the putamen and a decreased ADC in the pallidum and putamen were found [8]. While FA remained unchanged, we also observed decreased ADC in the putamen of patients with dystonia. No alterations were found for MTI in the basal ganglia, suggesting that detected grey matter volume changes are not due to severe macromolecular matrix alterations or cellular damage. According to a previous study [22], there was no correlation between disease duration or UDRS with volumetric alterations in the basal ganglia. However, longitudinal studies are necessary to answer the question of whether these volume changes are to be regarded as primary structural changes or secondary adaptations [24].

### Brainstem and cerebellum

Changes outside the basal ganglia can be interpreted to be a consequence of basal ganglia dysfunction. In a more recent view, dystonia can be regarded as a network disorder characterized by abnormal communication of any part of the network [25]. Among these networks, the cerebellum, cerebello-thalamo-cortical network and the pontine brainstem have received considerable attention [20,26,27]. A disruption of cerebellar output can be an important factor determining the occurrence of motor symptoms [27].

Evidence of abnormal interaction between the basal ganglia and cerebellar networks comes from structural and functional imaging studies and animal models. Our ROI analysis revealed cerebellar alterations in grey matter volume. Both increases and decreases in cerebellar grey matter volume were demonstrated before in VBM [3,4]. We also found altered MTI, diffusivity and FA values in the cerebellum of patients with dystonia. However, with regard to analysis of the cerebellum using DTI, several methodological limitations have to be taken into account, such as the low resolution after normalization and incomplete mapping of the cerebellum.

Decreased connectivity of the cerebellum with the thalamus was demonstrated by diffusion tractography in patients with clinically manifesting and non-manifesting hereditary dystonia [28]. Moreover, studies found increased metabolism in the cerebellum [28]. Animal models showing cerebellar dysfunction provide further support for the important role of the cerebellum in dystonia [27]. Our patients also exhibited DTI changes in the pontine brainstem, particularly the medial lemniscus, which carries sensory information from the gracile and cuneate nuclei to the thalamus. This is in line with DTI studies on hereditary dystonia, which revealed white matter changes in the dorsal pons [7,29]. The output from the basal ganglia to the brainstem targets regions that provide indirect input to the striatum [30]. It was postulated that these basal ganglia–brainstem pathways determine the degree of freedom of the automatic and volitional aspects of movements and are involved in the maintenance of arousal and attentive states and regulation of REM sleep [31].

### Somatosensory system and thalamus

Although dystonia is characterized by motor problems, sensory abnormalities for temporal discrimination, kinesthesia, perception of vibratory tendon stimulation and somatotopy were frequently observed by special testing [32]. One possible reason for these sensory abnormalities can be a loss of lateral inhibition [21]. Our study underlines the involvement of the sensory system. Decreased grey matter volume and white matter changes were found in the primary and secondary somatosensory cortices. The observed decrease in grey matter volume in the secondary somatosensory cortex is in line with previous findings in patients with blepharospasm [22]. Our study showed a decreased FA in the primary and secondary somatosensory cortices, which was not demonstrable in previous ROI-based studies [8,9] or in a study of patients with hereditary dystonia [7].

Correlation analyses revealed dependency of these microstructural changes on disease duration, suggesting that DTI changes reflect adaptation processes in the somatosensory system during the disease course or because of botulinum toxin treatment. This is supported by the observation that a decrease in grey matter volume in the parietal lobe depends on the duration of botulinum toxin treatment in patients with blepharospasm [22]. Likely, botulinum toxin leads to remodeling processes in the somatosensory system because of its local denervation in the muscle [33]. On the other hand, this toxin can mask morphological changes associated with dystonia [9,34]. To address this issue, a correlation analysis of the duration and dose of botulinum toxin should be performed.

DTI changes in the fibre tracts connecting the primary sensorimotor areas with the subcortical structures may be the basis for the impairment of GABAergic cortical inhibition proposed as a pathophysiological feature of dystonia [7,21]. Furthermore, changes in DTI in the thalamus were demonstrable, showing that the thalamus exhibits not only functional [35,36] but also structural alterations in patients with dystonia. Under the view of dystonia as a network disease, this result is not surprising because the basal ganglia not only contacts regions of the thalamus that project directly back to the basal ganglia input nuclei but also project back to those cortical regions providing original inputs to the striatum [30].

In summary, our results strongly support the thesis that the sensory system is frequently involved in dystonia [34,37-40]. In addition, this provides further evidence for the concept that abnormal motor learning or abnormal plasticity may play a crucial role in dystonia [19,21].

### Frontal areas

A decrease in grey matter and microstructural changes in white matter were observed in the supplementary motor area and primary motor cortex. Other prefrontal areas (BA 10) showed enhanced grey matter volume and decreased MTR. MTR was increased in the anterior cingulum of the patients with dystonia. While grey matter atrophy in the supplementary motor area has been previously demonstrated [3], this is the first time that an enhanced FA was observed in the supplementary motor area of patients with dystonia, supporting that these changes are not only functional [41,42] but also structural. The supplementary motor area is responsible for planning complex movements; therefore, it is connected with the primary motor cortex and the thalamus, both of which showed structural changes in this study. While a grey matter decrease in the supplementary motor area was not demonstrable in smaller cohorts of patients with dystonia [3] or blepharospasm [22], the decreased FA is in line with previous findings [7].

It seems reasonable that alterations in the dorsolateral prefrontal cortex can be observed in patients with dystonia because this structure is connected to the orbitofrontal cortex, thalamus, basal ganglia, hippocampus and primary and secondary association areas, including the posterior temporal, parietal and occipital areas. In a study of regional cerebral blood flow in 6 patients with idiopathic torsion dystonia, patients showed significant overactivity in the ipsilateral dorsolateral prefrontal cortex, demonstrating inappropriate activity of striatofrontal projections and impaired activity of motor executive areas [41]. Here, involvement of the dorsolateral prefrontal cortex could be demonstrated by DTI and MTI changes in the patients with dystonia and was supported by previous volumetric [3] and functional imaging studies [35,41]. With regard to the grey matter increase in the prefrontal areas (BA 10), it seems that the decreased MTR in this region is not caused by neuronal cell loss. The correlation between changes in the primary or secondary motor cortex and disease duration suggests that these are secondary adaptations, probably caused by modified thalamic firing patterns. This is supported by the positive correlation between the severity of dystonia (UDRS) and increase in grey matter volume in the frontal gyrus and cingulum.

### Visual system

Because the basal ganglia and motor areas are heavily connected with visual areas, one can assume that abnormalities in other sensory modalities, such as visual, may also be implicated in dystonia. We frequently observed changes in the lateral and medial surfaces of the occipital lobe, the frontal eye field and area MT with all three imaging methods. The frontal eye field in the patients with dystonia showed enhanced grey matter volume. In the secondary visual cortex, decreased MTR and FA and an increased diffusivity were found. In the visual areas, FA decreased (primary and secondary visual area, frontal eye field) and diffusivity increased (secondary visual cortex) with disease duration. With increasing severity of dystonia (UDRS), FA increased in the primary and secondary visual areas and the frontal eye field, whereas diffusivity decreased in the secondary visual cortex. Evidence for alterations in the visual system came from neuropsychological studies [43] and one volumetric analysis showing a grey matter decrease in the visual cortex [3]. The severity of dystonia (UDRS) correlated with increased FA in the primary and secondary cortices and decreased diffusivity in the secondary visual cortex. Disease duration correlated with decreased FA in the primary and secondary cortices and the frontal eye field. Moreover, duration correlated with increased diffusivity in the secondary visual cortex and decreased diffusivity in the frontal eye field. These results probably display the adaptation to the dystonic head shift and show some similarity to the grey matter volume increase in MT after juggling training [44].

The present study provides evidence that dystonia is a complex disturbance of neuronal circuits involving the basal ganglia, thalamus, motor cortex, premotor area and visual and frontal cortices.

### Methodological aspects and limitations of the study

This study is not free of limitations. The sample size is comparable with those in other studies on dystonia; however, analysis of more patients would be useful in order to study the impact of several clinical variables. Moreover, to understand the nature of primary and secondary processes, a longitudinal assessment of patients would be a challenge to expand the results obtained via correlation analysis.

As in many imaging studies, a primary limitation is that most data were uncorrected. Analyses using uncorrected voxel-based statistics have increased sensitivity to detect particularly small abnormalities, but the number of false abnormal regions is higher than that in analyses using corrected voxel-based statistics [45]. Because the usefulness of SVC described in the methods section is controversial, we also used ROI analyses with anatomically predefined regions for the group comparison; this supported and augmented the results from voxel-based imaging. In terms of DTI, both FA and ADC values were changed in the patients with dystonia. The value that accurately reflects white matter damage is not yet clear. Discrepancies in the DTI studies are primarily caused by differences in the number and age of the patients and severity and duration of the disease. One crucial factor is also the dataset. A dataset with more gradient directions was more sensitive to decreases in FA, while the dataset with more b values was more sensitive to increases in mean diffusivity [46]. Last, the results are influenced by the choice of MRI scanner (3-T or 1.5-T). A higher field strength at 3.0 T would probably have great impact on the results for DTI scalars and MTI.

## Conclusions

Despite the discrepancies in imaging results, some unifying conclusions can be drawn from the results of this study. VBM, DTI and MTI revealed widespread structural changes that were not restricted to the basal ganglia. Structural alterations were observed in the thalamus, motor cortex, premotor and frontal cortices, visual system and cerebellum of the patients with dystonia. This study provides further evidence that cervical dystonia is a multisystem disease that involves several networks such as the motor, sensory and visual systems.

## Abbreviations

BA: Brodman area; DTI: Diffusion tensor imaging; MRI: Magnetic resonance imaging; MTI: Magnetization transfer imaging; MTR: Magnetization transfer ratio; PD: Proton density; VBM: Voxel-based morphometry.

## Competing interests

The authors declare that they have no competing interests.

## Authors' contributions

Conceived and designed the experiments: ThP, JG, RD. Performed the experiments: ThP, JG, BK, MB, HB. Analyzed the data: ThP, JG, BK, TP, AG. Wrote the paper: TP. All authors read and approved the final manuscript.
